# Trim24 and Trim33 Play a Role in Epigenetic Silencing of Retroviruses in Embryonic Stem Cells

**DOI:** 10.3390/v12091015

**Published:** 2020-09-11

**Authors:** Liad Margalit, Carmit Strauss, Ayellet Tal, Sharon Schlesinger

**Affiliations:** Department of Animal Sciences, The Robert H. Smith Faculty of Agriculture, Food, and Environment, The Hebrew University of Jerusalem, Rehovot 7610001, Israel; liad.margalit@mail.huji.ac.il (L.M.); carmit.feliks@mail.huji.ac.il (C.S.); ayellet.tal@mail.huji.ac.il (A.T.)

**Keywords:** embryonic stem cells (ESC), endogenous retroviruses (ERVs), murine leukemia virus (MLV), epigenetic silencing, Tif1 family, Trim24, Trim28, Trim33

## Abstract

Embryonic stem cells (ESC) have the ability to epigenetically silence endogenous and exogenous retroviral sequences. Trim28 plays an important role in establishing this silencing, but less is known about the role other Trim proteins play. The Tif1 family is a sub-group of the Trim family, which possess histone binding ability in addition to the distinctive RING domain. Here, we have examined the interaction between three Tif1 family members, namely Trim24, Trim28 and Trim33, and their function in retroviral silencing. We identify a complex formed in ESC, comprised of these three proteins. We further show that when Trim33 is depleted, the complex collapses and silencing efficiency of both endogenous and exogenous sequences is reduced. Similar transcriptional activation takes place when Trim24 is depleted. Analysis of the H3K9me3 chromatin modification showed a decrease in this repressive mark, following both Trim24 and Trim33 depletion. As Trim28 is an identified binding partner of the H3K9 methyltransferase ESET, this further supports the involvement of Trim28 in the complex. The results presented here suggest that a complex of Tif1 family members, each of which possesses different specificity and efficiency, contributes to the silencing of retroviral sequences in ESC.

## 1. Introduction

Retroviruses can replicate in most tissues and cell types, and animal cells possess many regulatory pathways to restrain them, targeting almost all stages of the viral lifecycle [1]. Following entry, the retroviral genome is reverse transcribed and integrates into the host cell DNA to become a pro-virus. Endogenous retroviruses (ERVs) are the remnants of ancient infections of the host germline by retroviruses. ERV sequences have become a significant part, about 8–10%, of the mouse and human genome, respectively. Pluripotent cells, namely embryonic stem cells (ESC), possess the ability to restrain retroviral sequences, both exogenous and endogenous [2]. The predominant defense strategy of pluripotent cells is the repression of retroviral sequences’ transcription through heterochromatinization, which blocks the binding of transcriptional activators [3,4,5,6,7,8,9,10,11]. In many cases, a similar mechanism limits the expression of genes delivered by retroviral vectors [12] and is responsible for both exogenous and ERV silencing [3,6,11].

TRIM28 is a KRAB associated protein (KAP1) and a transcriptional intermediary factor 1 family member (TIF1β) [13,14]. TRIM28 forms a dimer [15,16,17,18] that recruits the H3K9 lysine methyltransferase SETDB1/ESET to the murine leukemia virus (MLV) provirus and various ERVs [4,7,11] and mark them with H3K9 tri-methylation [10,19,20]. SETDB1, but also H3K9me3 readers (e.g., heterochromatin protein HP1), the ATP-dependent chromatin remodeler SMARCAD1 [21] and effector proteins required for the formation and expansion of heterochromatin (reviewed in [22,23]) are pivotal for ERV silencing. The Histone chaperones Chaf1a and b, the sumoylation factor Sumo2 and the H3.3 chaperones ATRX and DAXX were also shown to regulate ERVs in a Trim28-dependent manner [24,25,26]. Trim28 binds the majority of evolutionary young KRAB-ZFPs, many of which have binding sequences in particular families, both in mice and in humans [8,9,27,28,29]. In MLV, the repression is partially dependent on a conserved sequence element termed the primer binding site (PBS). This is an 18-nucleotide sequence complementary to the 3′ end of proline tRNA, the tRNA primer used for initiation of reverse transcription by MLV [30,31]. Silencing is mediated by ZFP809, which binds exclusively to the proline PBS (PBS-P) sequence of the integrated provirus, and Trim28, which interacts with ZFP809 [4,5]. However, retroviral vectors utilizing alternative PBS sequences (Glutamine, for example–PBS-Q), which are not recognized by this silencing machinery, are still silenced by Trim28 [6,11,32,33]. This silencing might be reinforced by factors such as YY1 [34], EPB1 [35] and the HUSH complex [36], which were shown to interact and assist Trim28 binding to the viral long terminal repeats (LTR) region. Overall, Trim28 can be recruited through a multitude of different site-specific DNA binding proteins to more than one region within an ERV sequence, to fully condense and remodel the proviral chromatin to heterochromatin. Hence, proviral sites are silenced due to the Trim28 complex chromatin repression-remodeling activity. However, less is known about the targeting of Trim28 to the retroviral sequences, and even less understood is the cause for the specific action of this complex in specific cell types, like ESC.

The transcriptional intermediary factor 1 (TIF1) family proteins Trim24/Tif1α, Trim28/Tif1β/KAP1, and Trim33/Tif1γ/ectodermin are characterized by an N-terminal tripartite motif (TRIM) domain consisting of a RING domain, two B boxes and a coiled-coil, and a C-terminal PHD finger and bromodomain [37]. They are the only TRIM proteins with these histone-binding domains and were implicated in tumorigenesis, heterochromatin formation, and DNA repair, amongst other functions. Tif1 family members cannot directly bind to DNA but, depending on the cellular and signaling context, can be recruited by cell type-specific transcription factors to act as a transcriptional co-activator or co-repressor [38]. Additionally, like many TRIM proteins, the Tif1 family members are ubiquitin E3 ligases, possibly also for ubiquitin-like molecules, such as SUMO [39]. Despite their similar overall structure, these proteins have diverse functional roles in transcriptional regulation. Trim24 is a ligand-dependent nuclear receptor coregulator and has been implicated in regulating p53 stability [40] and cytokine expression in Th2 cells. Trim28, other than repressing retroviral transcription, functions as a SUMO ligase, sumoylating the adjacent bromodomain [38], in a ubiquitous chromatin remodeler and is important for DNA break repair [41]. Trim33 does not interact with HP1 or chromatin-remodeling/modifying complexes, but functions in the TGF-β superfamily pathways [42,43,44].

Here, we study the involvement of the Tif1 family members, namely Trim24 and Trim33, in retroviral silencing. In the murine liver, these three Tif1 proteins were shown to form a regulatory complex that suppresses hepatocellular carcinoma [45,46]. Together, these factors inhibit a member of the ERV1 class, VL30, and thus play an important role in retroviral restriction [47]. Interestingly, some VL30 elements possess PBS-P sequence and are silenced by ZFP809 [48]. Furthermore, Trim33 functions as a transcriptional repressor of RLTR10B—the LTR of the ERV class II (also known as ERVK) MMERVK10C elements—in the testis [49]. Interestingly, Trim33 also plays an important role in HIV-1 replication and proviral DNA formation [50].

## 2. Materials and Methods

### 2.1. Cell Culture and Differentiation

NIH3T3 and HEK293 cells were cultured in standard conditions (Dulbecco modified Eagle medium (DMEM) with 10% FBS, 2 mmol/L L-glutamine, 1000 U/mL penicillin, 100 mg/mL streptomycin). R1-ESCs were cultured on gelatinized tissue culture plates in ESC media (DMEM supplemented with 15% defined fetal bovine serum (HyClone Cat. SH30070), 100 IU/mL penicillin, 100 mg/mL, streptomycin, 2 mmol/L L-glutamine, 5 mg/mL MEM non-essential amino acids, 1 mM sodium pyruvate, 0.12 mmol/L b-mercaptoethanol, and 1000 U/mL leukemia inhibitory factor (LIF, (peprotech)) supplemented with the 2i inhibitors cocktail (mitogen activated protein kinase (MAPK)/extracellular signal-regulated kinase (ERK) kinase (MEK) inhibitor PD0325901 and the glycogen synthase kinase 3 (GSK3) inhibitor CHIR99021). All cells were cultured at 37 °C in 5% CO2. Retinoic acid (RA) induction was done in 1uM concentration in ESC media without LIF and 2i.

### 2.2. Stable shRNA and Overexpression Cell Line Production and Transduction

shRNA KD was performed as in Schlesinger and Goff [3], using pLKO.1 (Sigma, Rehovot, Israel) based plasmid. Retroviruses for transduction assays were prepared as described using lentiviral vectors. VSV-G pseudotyped viruses were generated by co-transfection of 293T cells with a mixture of the vector and the helper plasmids, pCMV-intron and pMD.G, using a 10:9:1 ratio of the three plasmids in each transfection. All viruses were harvested 48 h post-transfection, filtered (0.45 mm filter, Millipore, Burlington, MA, USA), aliquoted, and stored at −80 °C. Neat medium containing retroviruses (with 8 mg/mL polybrene) was used to infect cells that were then allowed to recover for 48 h followed by selection with 20 mg/mL puromycin. For viral transduction assays, viruses were prepared as above using pNCA-GFP vectors [34]. Retroviral stocks were then serially diluted (for titer determination), and infection efficiency was measured by flow analysis of the NIH3T3 cells 2 days after infection. Each experiment was repeated as indicated.

### 2.3. Co-Immunoprecipitation

For whole cell extraction, trypsinized cells were washed with PBS and resuspended in ice-cold hypotonic lysis buffer (20 mM Tris-HCl pH 7.5, 0.2 mM ethylenediaminetetraacetic acid (EDTA), 0.5 mM 1,4-dithiothreitol (DTT), 1mM NaVO4, protease inhibitors cocktail 1:100 [Sigma]). Cells were incubated for 10 min on ice. A similar volume of high salt buffer (20 mM Tris–Cl pH 7.5, 0.2 mM EDTA, 0.5 mM DTT, 1 M NaCl) was added and cells were incubated 15 min on ice. Cells were then centrifuged (20,000× *g*, 4 °C, 30 min) and the supernatants were collected for further analysis. Extracts were diluted with immunoprecipitation (IP) wash buffer (20 mM Tris-HCl pH 7.5, 0.2 mM EDTA, 0.5 mM DTT, 0.2% Triton X-100, 150 mM NaCl) to a final volume of 400 µL. Four micrograms of antibody were added to the lysate and incubated overnight in 4 °C, with rotation. Beads (Dynabeads; Invitrogen, Carlsbad, CA, USA) were magnetically pulled down as recommended by manufacturer and washed 3 times with ice-cold IP wash buffer. After the final wash, beads were incubated with 2x SDS loading buffer, heated at 95 °C for 3 min, and the supernatant was collected and observed by Western blot. Antibodies used for Pulldown and Western blot were anti-Trim28 (ab22553, Abcam, Cambridge Britain), anti-Trim24 (A300-815A, Bethyl, Montgomery, TX, USA), anti-Trim33 (ab47062, Abcam), anti-IgG (SC-69916, Santa Cruz, Dallas, TX, USA), anti b-Actin (47778, Santa Cruz), anti GAPDH (25778, Santa Cruz). Detection for Western blot was with anti-mouse or anti-rabbit antibodies conjugated to HRP (115-035-062, 111-035-144 respectively, Jackson ImmunoResearch, Pennsylvania, USA). Quantification of WB band intensity was done using ImageJ, and the values shown are normalized to the WT sample.

### 2.4. RNA Extraction, Reverse Transcription and Real-Time PCR

RNA extraction from cells was carried out using PureLink RNA Minikit (Invitrogen, Carlsbad, CA, USA). RNA was then reverse-transcribed into cDNA using High-Capacity cDNA Reverse transcription kit (Applied Biosystems, Foster City, CA, USA). Real-Time PCR (RT-qPCR) reactions were performed using Fast SYBR Green Master Mix (Applied Biosystems,) in an ABI Step-One Plus Real-Time PCR system. To ensure validity, each sample tested in triplicates (technical replicates). All primers used were tested and found agreeable with standard curve evaluation. The relative mRNA fold change was calculated with the ΔΔct method using *UBC* control gene as reference and WT-ESC expression values. For primer sequences, see Table S1.

### 2.5. Chromatin Immunoprecipitation

Chromatin immunoprecipitation (ChIP) was performed using 5 × 10^6^ cells that were crosslinked with 1% formaldehyde at room temperature for 10 min. Chromatin was extracted and then sonicated to an average size of 300 bp. Immunoprecipitation was carried out by using Magna ChIP ™ kit (Millipore,) and DNA was purified using QIAquick PCR purification kit (Qiagen, Hilden, Germany). Anti-trimethyl-histone H3 (Lys9) (07-442, Millipore,) was used. IgG antibody was used as a negative control (sc-2027, Santa Cruz,). Amplification was carried out by real-time PCR, and the bound/input values were then normalized by setting the negative control gene results to 1. For primer sequences see Table S1.

### 2.6. Flow Cytometry

Cells were detached from the plate using trypsin solution then filtered through a 35uM nylon filter (Thermo Fisher, Waltham, MA, USA). The fluorescence of the cells was determined using the Flow Cytometer Cytoflex from Beckman Coulter, equipped with 488 nm laser. A minimum of 10,000 cells were examined for each measurement at a flow rate of 12–35 μL/s. Cell debris was excluded from the analysis utilizing gating based on cell complexity and cell size (SSC-A and FSC-A respectively) parameters. Flow cytometry results were analyzed using FlowJo V.10 software. Normalization to the infection efficiency as seen by the percentage of NIH 3T3 expressing cells to correct for variation in multiplicity with different viruses was done. The numbers are given as GFP-positive ESC/GFP-positive NIH3T3 cells ×100.

### 2.7. Bisulfite Analysis

Bisulfite conversion of genomic DNA was carried out using Qiagen EpiTect Bisulfite Kit (Hilden, Germany) according to the manufacturer’s instructions. PCR primers were used as in [3]. After PCR amplification, products were extracted from gels using the Qiagen MinElute Gel Extraction Kit, cloned into the Promega (Madison Wisconsin, USA) pGEM-T vector system, transfected into Promega JM109 competent bacteria, and plated on LB-Agar + Amp + X-gal and IPTG. The DNA from 10–20 white clones of each ligation was subjected to sequencing with T7 or Sp6 primers. Sequence results were analyzed with the BiQ analyzer software [51], using all standard quality control steps.

## 3. Results

### 3.1. Tif1 Family Members Are Co-Expressed and Bind to Each Other in ESCs

**Figure 1:**
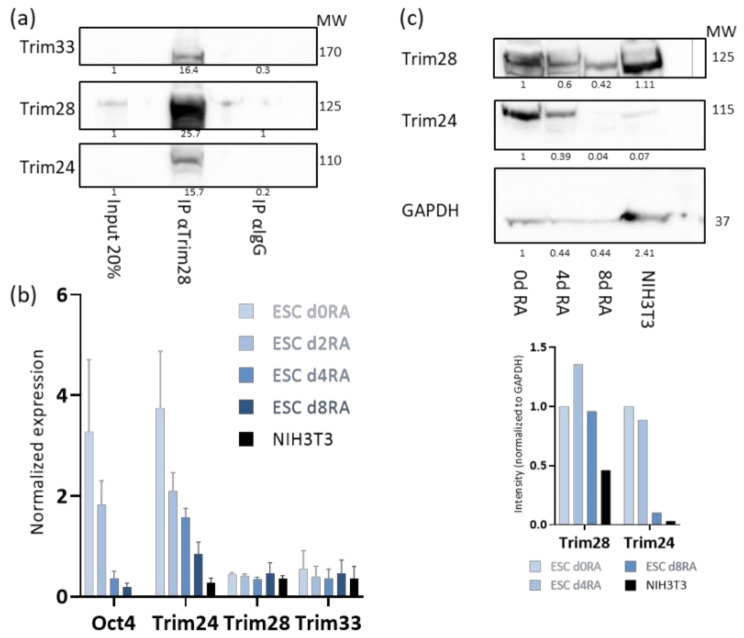
Tif1 family members are co-expressed and bind to each other in embryonic stem cells (ESCs). (**a**) Whole-cell protein extract co-immune precipitation (co-IP) and Western blot analysis with the indicated antibodies; (**b**) ESCs were grown with retinoic acid RA and no 2i/Lif for the indicated times, RNA expression levels of the indicated genes were measured by RT-qPCR and normalized to *UBC* control. NIH3T3 fibroblasts are used as differentiated control *n* = 3; (**c**) protein extract from ESCs treated with RA for 0, 4 or 8 days, and NIH3T3 cells were subjected to western blot analysis (top), showing downregulation of Trim24 protein levels after differentiation onset. Normalized density measurements are displayed (bottom).

The Tif1 family members, Trim24, Trim33, and Trim28, are expressed in ESCs. To test if they form complex, co-immune precipitation (co-IP) assay was performed using whole-cell extract from ESC. Trim28 pulled down Trim24 and Trim33 (Figure 1a) and Trim24 pulled down Trim28 and Trim33 (Figure S1). α-IgG antibody was used as a negative control. Next, we wondered if the presence of this protein complex is unique to the undifferentiated state of ESC. It is known that Trim28 is expressed in differentiated as well as undifferentiated cells. The expression levels of *Trim24* and *Trim33* was measured in ESC in the presence of RA, which induces differentiation. *Trim24* RNA expression decreases upon 4 days of RA, while *Trim28* and *Trim33* RNA expression levels are not changed (Figure 1b). The decrease in Trim24 in differentiated cells was also verified at the protein level both in RA treated ESC and NIH3T3 cells (Figure 1c).

**Figure 2:**
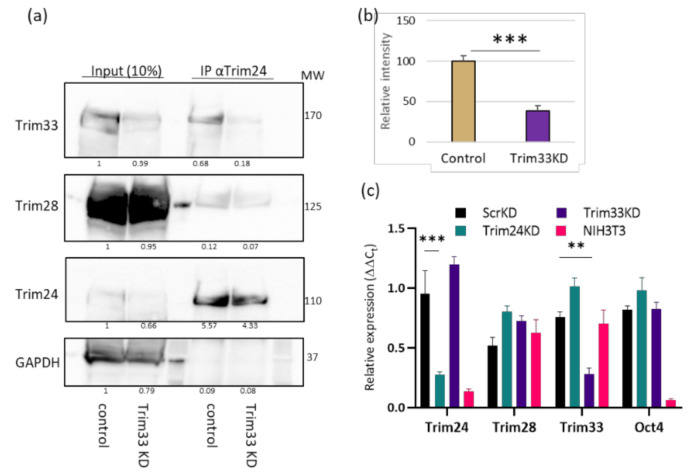
The depletion of Trim33 disrupts the binding of Trim24 to Trim28. (**a**) Whole-cell protein extract was generated from control or Trim33 KD ESCs and subjected to co-IP assay using anti Trim24 antibody flowed by Western blot analysis. Binding between Trim28 and Trim24 was measured by the bend intensity of Trim28/Trim24 in the coIP lanes. (**b**) A statistical representation of 3 biological repeats of the experiment described in (**a**) Student’s t-test *p* < 0.001; *n* = 3. (**c**). RT-qPCR expression analysis to verify depletion in KD cells normalized to UBC control gene and WT ESC cells. No change was observed in Trim28 and the Oct4 pluripotency gene expression. The values are averages of three or more independent experiments ± SEM. ** *p* < 0.01; *** *p* < 0.001.

To validate that Trim33 is required in the complex, we examined if the interaction between Trim24 and Trim28 is Trim33-dependent. *Trim33* was downregulated by expression of shRNA. Control and Trim33 KD cells were subjected to co-IP using α-Trim24 antibody followed by Western blot. KD of Trim33 impairs the interaction between Trim28 and Trim24 and lowers it to 38% of the control (Figure 2a,b). These results suggest that the Tif1 family members form a complex together in ESC. Since the involvement of Trim28 in silencing retroviruses in ESC is well established, we set out to explore whether Trim24 and Trim33 are also involved in this process. To this end, *Trim24* or *Trim33* were depleted in ESC by specific shRNAs stable transduction. RNA was extracted from the KD and control cells, and knockdown was verified using RT-qPCR (Figure 2c). The depletion did not affect the expression levels of the other TIF1 genes or on the pluripotency of the cells, measured by *Oct4* expression levels.

### 3.2. The Tif1 Complex Contribute to the Silencing of Some ERVs Subfamilies

**Figure 3:**
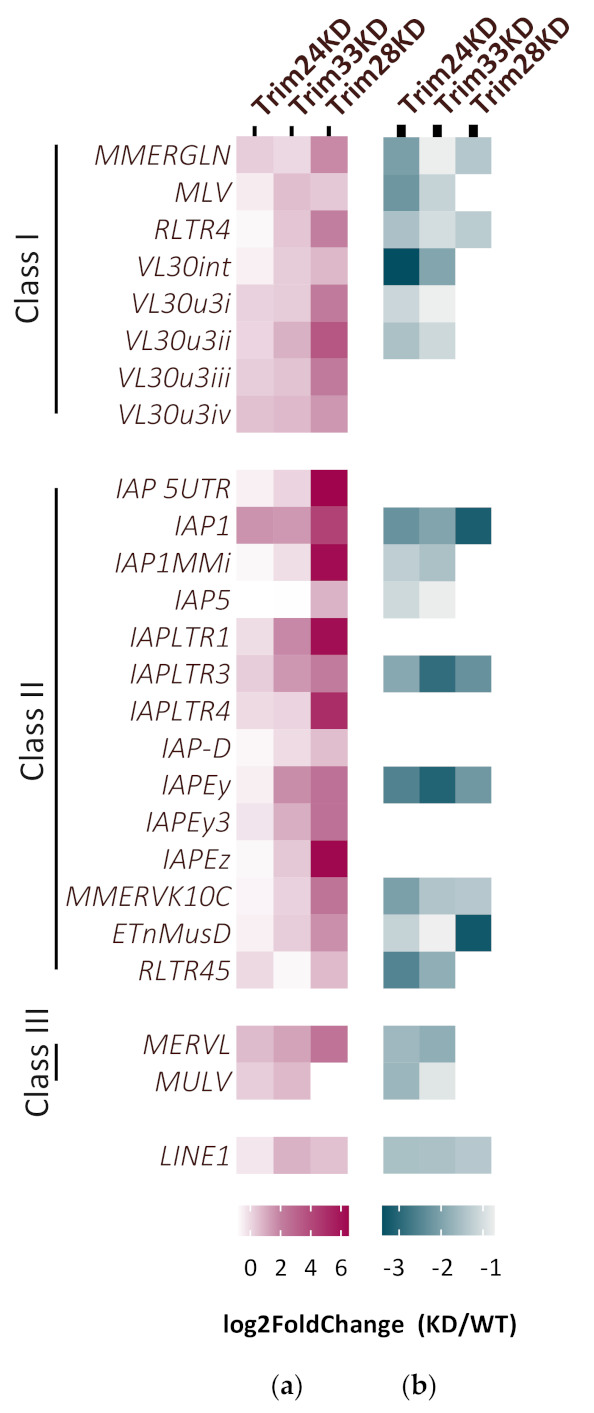
Tif1 family members contribute to the silencing of distinct ERVs subfamilies. (**a**) ERV expression change of KD vs. WT was measured using RT-qPCR. The three KD are ESCs expressing shRNA targeting either Trim24, Trim33 or Trim28 (*n* > 3). (**b**) H3K9me3 enrichment on ERVs was measured by chromatin immunoprecipitation (ChIP). Normalized log2 fold change of KD vs. WT is presented (*n* = 2) Statistical significance determined using the Holm-Sidak method, with alpha = 0.05, see also Figure S3.

To test the involvement of Trim24 and Trim33 in ERV silencing, RNA levels of representative ERV family members were examined in the KD cells. Elevated expression of some ERVs in the KD cells over the control is observed, especially following Trim33 depletion (Figure 3a). Interestingly, the two KD had a different and specific effect on the various ERV families. While Trim33 KD induces the expression of a similar set of ERVs like Trim28 KD, Trim24 seems to be responsible for the repression of other ERV subfamilies. Trim28 represses ERV expression by recruitment of the histone lysine methyltransferase ESET and the establishment of heterochromatin formation on the viral sequences [11]. We hypothesize that without the Tif1 complex formation, this epigenetic silencing might be compromised. Thus, ChIP was performed on the KD cells using α-H3K9me3 antibody, followed by RT-qPCR with ERV family primers. Decreased levels of H3K9me3 was detected on some ERVs in line with the change in their expression levels (Figure 3b). These data show that an increase in ERVs expression following downregulation of Trim24 or Trim33 is in correlation with a decrease in the chromatin modification H3K9me3 on these sequences. Detailed results of RT-qPCR and CHIP are presented in Figure S2a,b.

### 3.3. Decrease in Expression and Epigenetic Silencing of Pro-Viral Sequences Following Trim24 or Trim33 Depletion

**Figure 4:**
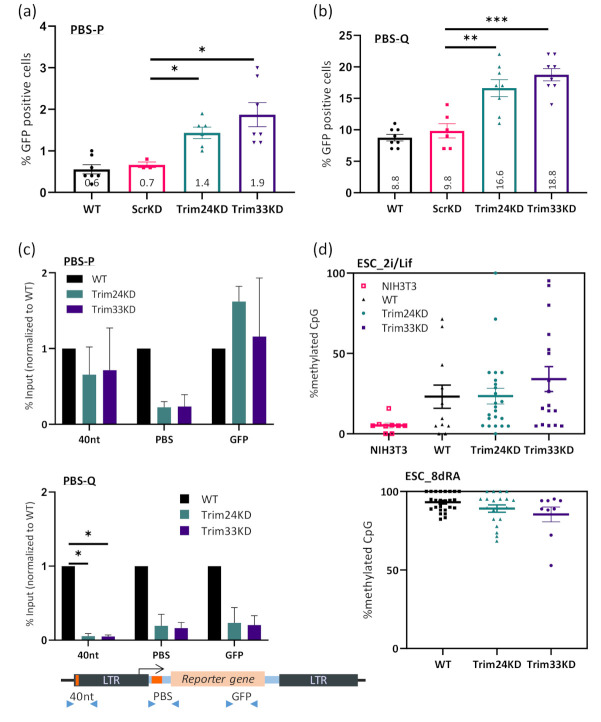
An increase in expression and a decrease in epigenetic silencing of pro-viral sequences following Trim24 or Trim33 depletion. Flow analysis of ESCs expressing shRNA targeting either Scr, Trim24, Trim33 or none, infected by exogenous murine leukemia virus (MLV) vector containing a GFP reporter, with either PBS-P (**a**) or PBS-Q (**b**). The percentage of cells expressing the GFP reporter was measured and normalized to the expression levels in NIH3T3 cells (see Figure S4). Statistics analysis by two-tailed Student’s t-test; * = *p* < 0.05; ** = *p* < 0.01; *** = *p* < 0.001; *n* > 5. (**c**) ChIP followed by RT-qPCR using H3K9me3 antibody and primers for pro-viral sequences. The ChIP was done separately on cells infected by either PBS-P (top) or PBS-Q (bottom). The location of primers on the proviral genome is illustrated in the diagram below. See Figure S3 for ChIP controls, statistical analysis was performed by two-tailed student t-test; * = *p* < 0.05; *n* = 2. (**d**) Bisulfite sequencing analysis of the 5′ LTR–the 40nt region-of the infecting virus was performed on WT, Trim24 KD and Trim33 KD ESC and NIH3T3 differentiated cells as control; ESC was next differentiated by 8 days RA induction and methylation status was re-examined. Percentages of methylated CpGs are shown for 10–20 cloned DNA molecules per cell (see Figure S5).

Following the finding that both Trim24 and Trim33 play a role in ERVs silencing, we tested their contribution to exogenous MLV silencing. We infected the cells with virus-like particles containing MLV viral genome expressing GFP reporter and utilizing either the wild type PBS-P (proline) or a variant PBS-Q (glutamine) as described previously in [34]. GFP expression in the cells was measured using flow cytometry 2–4 days after infection. The PBS-P and PBS-Q viruses were both efficiently expressed in differentiated NIH3T3 cells but were silenced in ESC (Figure 4a,b), although the PBS-Q virus was only partially silenced in these cells, as shown before. In the cells in which expression of Trim24 or Trim33 was suppressed, silencing was impaired. A two-fold increase in the expression of PBS-P and PBS-Q was observed. Like for the ERVs, the Trim33 KD seemed to have a slightly higher impact on the silencing. Viral load and proviral copy number in the cells is presented in supplementary Figure S3a,b. Exogenous retroviral repression is also mediated by epigenetic modifications such as H3K9me3 [3,4]. Therefore, ChIP was performed using α-H3K9me3 antibody on ESCs control, Trim24 KD, Trim33 KD, and NIH3T3. H3K9me3 histone mark levels at around the TSS (PBS) were lower in the Trim24 KD and Trim33 KD compared to ESCs (Figure 4c). While in the PBS-Q, both upstream and downstream locations were affected by the KD, no change was observed in H3K9me3 levels at the PBS-P on other sites. This could be explained by the highly efficient and specific binding of the silencing complex to the PBS-P region through ZFP809. Notably, the effect of the silencing complex binding on the PBS-Q virus is both less effective (~2% vs. 10% cells expressing the virus) and more diffused, expanding from the 5’UTR of the viruses and up to the coding region. Last, we wished to explore the possible effect of Trim24 and Trim33 depletion on the proviral DNA methylation. Naïve ESCs presumably mimic the “ground-state” conditions of the ICM when grown using ‘2i’ media (containing GSK3β and Mek 1/2 inhibitors), and do not have DNA methylation machinery [52]. However, some sequences, especially repeats, transposable elements, and ERVs, retain low levels of methylated CpG [53]. Therefore, we wished to examine the ability of these cells to methylate incoming MLV integrants, in WT and KD cells. Interestingly, the proviral sequence showed some level of CpG methylation in all the cells, regardless of the KD (Figure 4d, upper panel and Figure S4). Thus, we asked whether the decrease in H3K9me3 chromatin mark will affect the levels of DNA methylation following differentiation onset. After 8 days of RA treatment, methylation levels of all proviral sequences reached almost 100%, without a significant difference between the KD cells and the WT Figure 4d, lower panel and Figure S4). No difference was observed between the PBS-P and the PBS-Q sequences, and it should be noted that the levels of KD were not examined after the induced differentiation.

## 4. Discussion

In this study, a new role is shown for the Tif1 family members’ complex, namely Trim24, Trim28, and Trim33, in retroviral silencing. In mouse embryonic stem cells (ESCs), MLV exogenous retroviruses and many ERV families are known to be subjected to transcriptional silencing, mediated by heterochromatin formation and silencing epigenetic modifications. We show here that in ESCs, the three Tif1 family members are expressed and form a complex, which is dependent on the presence of Trim33. Technical issues prevented us from depleting and pooling down the other members, but we hypothesize that all three proteins are needed and therefore, when cells begin differentiation and Trim24 is no longer expressed, or when we deplete Trim33 expression by shRNA, the complex can no longer form.

Next, we show that in ESCs, the complex is essential for full repression of some ERV subfamilies, and depletion of one member of the Tif1 family compromises the silencing and enhances the expression of those ERVs. This upregulation is accompanied and probably resulted from a decrease in the silencing chromatin modification H3K9me3, which is responsible for the heterochromatin formation and silencing on those ERVs in WT ESC. While we show fine anti-correlation between expression and H3K9me3 enrichment, it should be noted that the different KD ESCs upregulate different ERV members—while Trim33 KD affects mostly class II ERVs (IAP family members), Trim24 represses the VL30 ERVs and others, but less on the IAP family. Whereas Trim28 depletion results in the overall upregulation of almost all ERVs. Hence, the notion emerging is that Trim28 and Trim33 show similar repression patterns, while Trim24 is more unique. These data suggest that while general mechanisms can be identified, each ERV sub-family is regulated autonomously, as is supported by the reported changes of expression during early development [54].

Similar to ERVs, MLV like exogenous retroviruses silencing also requires the presence of all three Tif1 family proteins for repression. Interestingly, the presence of the PBS-P sequence used for the ZFP809 binding site [5] alleviates the need for the complex, while in the PBS-Q type proviral sequence the upregulation following Trim24 or Trim33 depletion is more pronounced.

**Figure 5:**
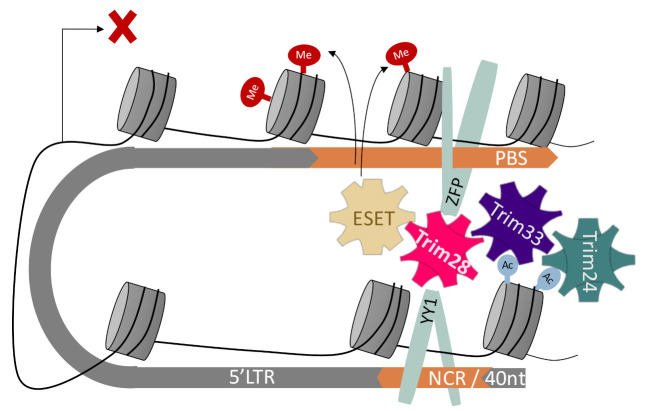
Model for Tif1 family members’ complex formation. A suggested interpretation of the data, showing binding of Trim24 and Trim33 to areas distal from the transcription start site and the PBS. By modifying the 3D structure of the provirus, the complex can be formed or collapse in a dynamic manner.

Trim33 PHD finger-bromodomain is a chromatin-binding reader module with specificity for acetylated H3 tail, and the histone binding activates its E3 ligase activity [55]. Trim24 has a similar preference to acetylated histones, which leads to its SUMOylation [56]. Thus, Trim24 and Trim33 can both bind to acetylated, enhancer-like sequences and function as dynamic co-repressors, as suggested by [55,57]. However, ERV elements in ESC are usually regarded as “heterochromatinised” and strictly silenced, raising the question–why and how do those proteins bind them? Based on our data, we can suggest the formation of a multimeric Tif1 complex that is functioning as a retroviral silencing complex, as illustrated in Figure 5. However, it will be interesting to examine the acetylation status of the ERV bound by Trim24 and Trim33. It could be hypothesized that by targeting the more open and dynamic ERVs, the ones who are still acetylated, this complex is functioning as the first line of defense against retroviral activation in pluripotent stem cells.

## 5. Conclusions

We demonstrated the formation of a complex by the three Tif1 family members-Trim24, Trim28, and Trim33, and established their function as repressors of retroviral transcription.

## Supplementary Materials

The following are available online at https://www.mdpi.com/1999-4915/12/9/1015/s1, Figure S1: **IP with anti Trim24**; Figure S2: RT-qPCR and ChIP on ERVs; Figure S3: NIH3T3 control and copy number of MLV infection; Figure S4: Bisulfite analysis of proviral DNA; Table S1: Primers list
