# Supplementary material for: Trim24 and Trim33 Play a Role in Epigenetic Silencing of Retroviruses in Embryonic Stem Cells
Source: Viruses. 2020 Sep 11;12(9):1015. doi: 10.3390/v12091015 (PMC7551373; doi:10.3390/v12091015)
Supplement: Supplementary file 1 [file viruses-12-01015-s001.pdf]

Supplementary figure S1

(a)

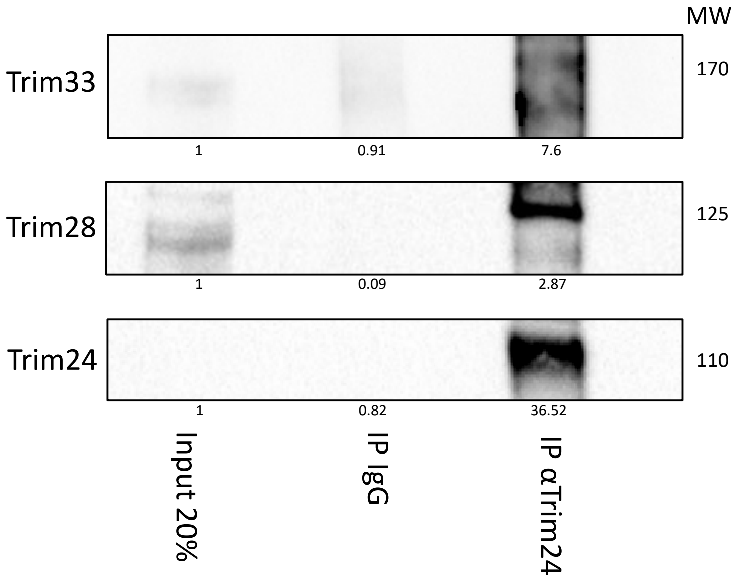

Supplementary Figure S1. IP with anti Trim24
Whole cell protein extract was generated from ESCs and subjected to co-IP assay flowed by western blot analysis. Antibodies are indicated in figure.

Supplementary figure S2

(a) Detailed results of RT-qPCR in fig 3a

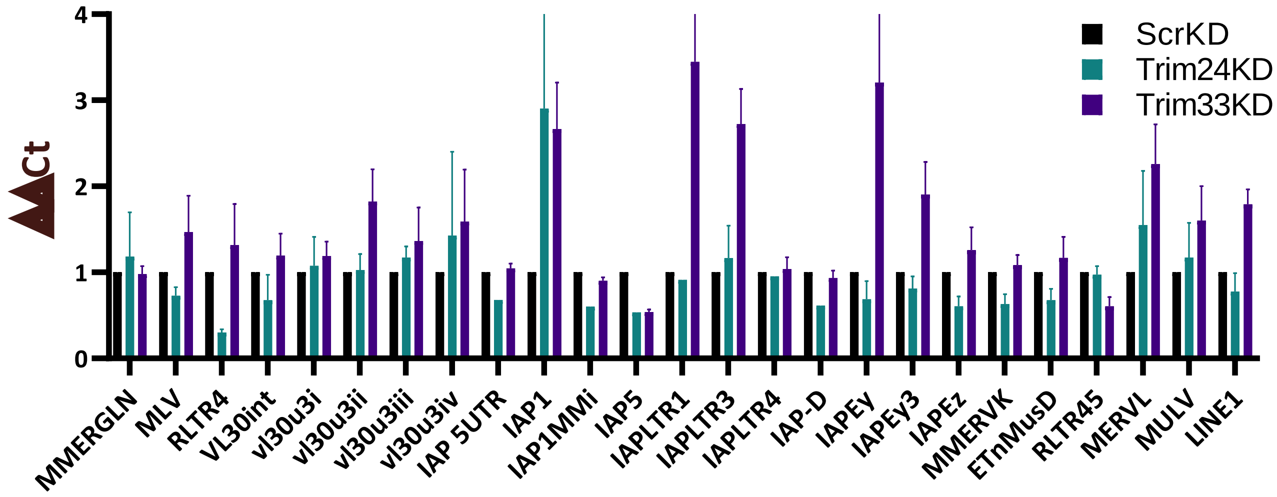

(b) Detailed results of ChIP in fig 3b

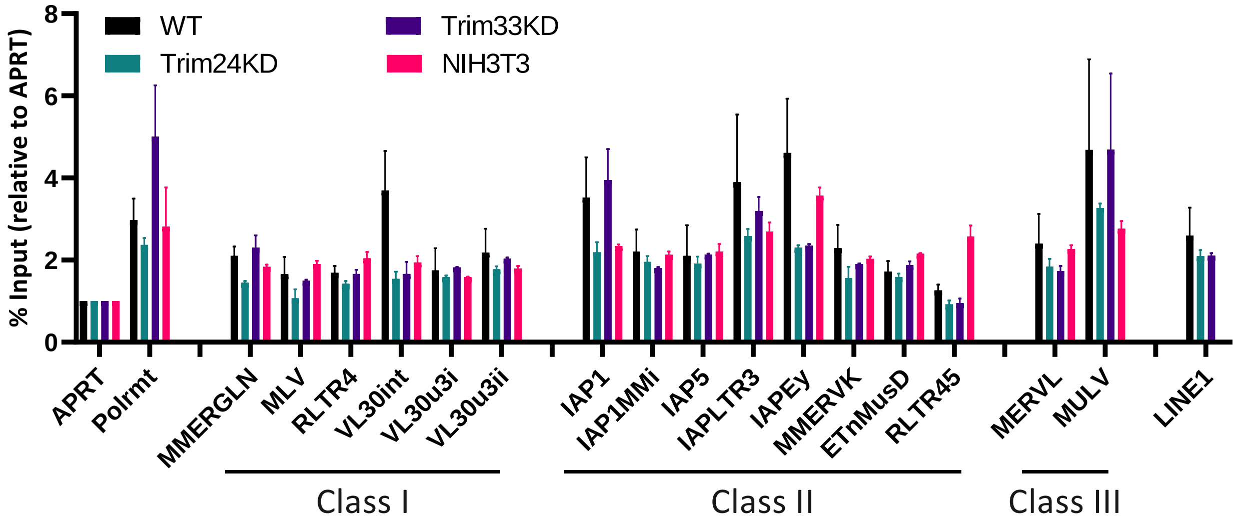

Supplementary Figure S2. RT-qPCR and ChIP on ERVs

(a) ERV RNA expression was measured using RT-qPCR on ESCs expressing shRNA targeting either Trim24, Trim33 or Scrambled sequence as control. Ct values were normalized to UBC control gene and to WT ESC. Control PCR reactions lacking cDNA (no RT) were below detection in all cases. (N>3) **(b)** ChIP-based measurement of H3K9me3 with primers specific for different transposable elements, mainly ERVs. Positive control gene (Polrmt) gave the expected results. Graphs show the mean enrichment ± SEM from two independent experiments relative to the total input samples and normalized to the signal of negative control (Aprt). Statistical significance determined using the Holm-Sidak method, with alpha = 0.05. The results are summarized in Figure 3.

Supplementary figure S3

(a)

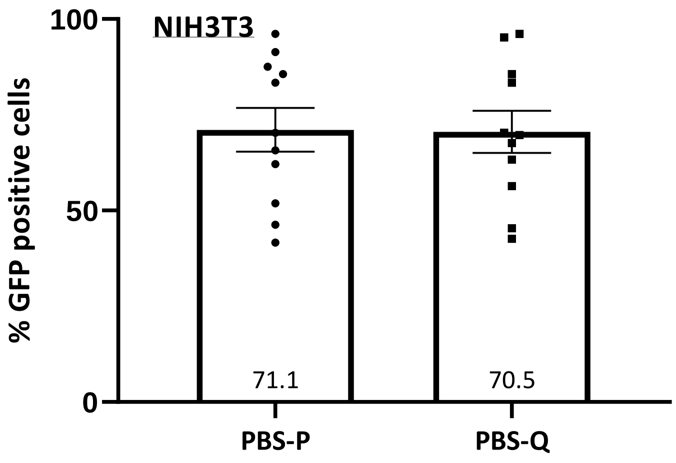

(b)

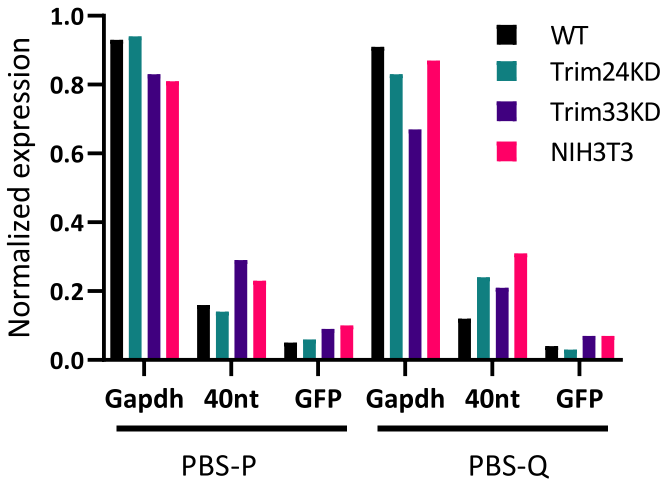

Supplementary Figure S3. NIH3T3 control and copy number of MLV infection
(a) NIH3T3 cells infected by exogenous MLV containing a GFP reporter, either PBS-P or PBS-Q. Percentage of cell expressing the GFP reporter was measured using a flow cytometer. This results were used to normalize GFP expression for the results described in Figure 4a.
(b) Analysis of proviral DNA copy number in the indicated cell lines as determined by qPCR.

Supplementary figure S4

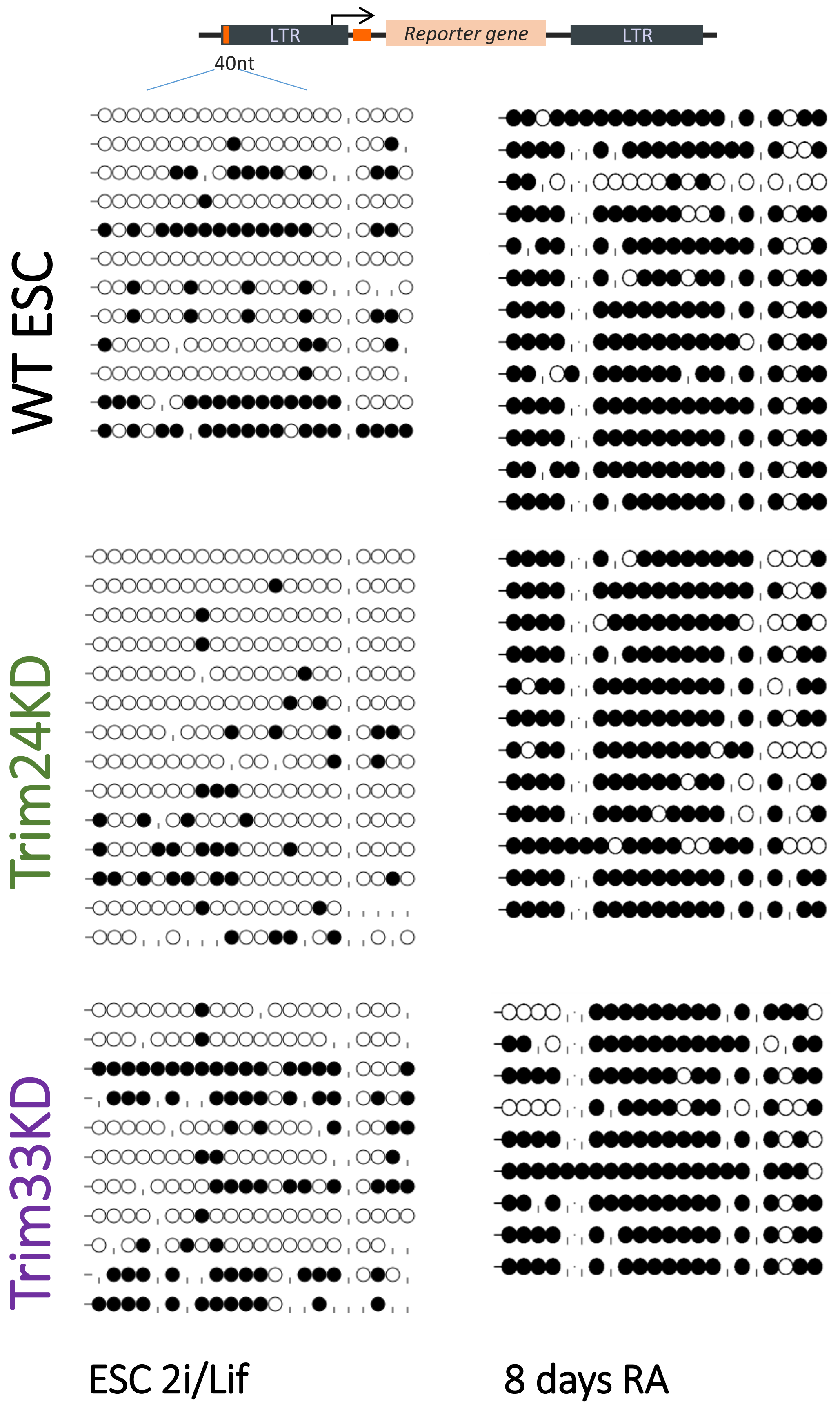

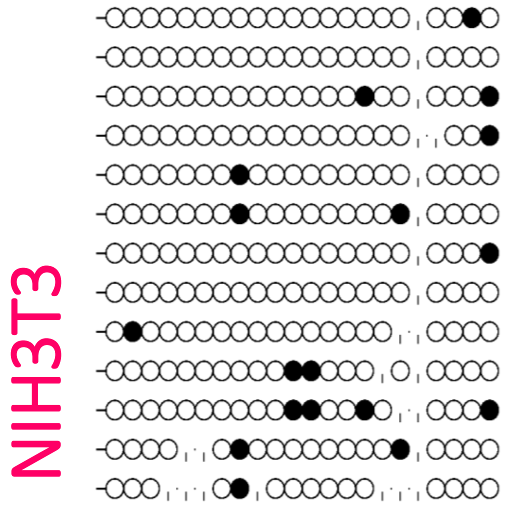

Supplementary Figure S4. Bisulfite analysis of proviral DNA

Bisulfite analysis was carried out on the unique 40nt region of the MLV vector, and the results of individual molecules are shown for WT and KD ESC lines, and differentiated NIH3T3 cells as control. The map indicates
the relative position of the tested sites. The results are summarized in Figure 4c.

Table S1: Primer list:

Target	F/R	sequence	application
UBC	F	CAGCCGTATATCTTCCCAGACT	RT-qPCR
UBC	R	CTCAGAGGGATGCCAGTAATCTA	RT-qPCR
Oct4	F	AACCAACTCCCGAGGAGTCCCA	RT-qPCR
Oct4	R	TCTTCTGCTTCAGCAGCTTGGCA	RT-qPCR
Trim24	F	CTGCACCAGCTTGAGAGTCTT	RT-qPCR
Trim24	R	AGCCACTTCCTGCTGTTGTT	RT-qPCR
Trim28	F	CTGCACCAGCTTGAGAGTCTT	RT-qPCR
Trim28	R	AGCCACTTCCTGCTGTTGTT	RT-qPCR
Trim33	F	CAGGTGAAGGTCAAGCAGGA	RT-qPCR
Trim33	R	AGGACTGCTCAACATACAGGC	RT-qPCR
Polrmt	F	AGACACCTGCTGCCCTATGT	ChIP
Polrmt	R	GCTCCATCCCAGTGCTTTAC	ChIP
APRT	F	GGGATATCTCGCCCCTCTT	ChIP/Copy number
APRT	R	CACTCGCCTGCGATGTAGT	ChIP/Copy number
Gapdh	F	ATCCTGTAGGCCAGGTGATG	ChIP/Copy number
Gapdh	R	AGGCTCAAGGGCTTTTAAGG	ChIP/Copy number
40nt LTR	F	CCTCCACACACCATCACTCA	ChIP/Copy number
40nt LTR	R	CCTGTTTGGCCCATATTCAG	ChIP/Copy number
PBS_MLV	F	TGGCCAGCAACTTATCTGTG	ChIP/Copy number
PBS_MLV	R	CAGGCGCATAAAATCAGTCA	ChIP/Copy number
GFP	F	AGCTGAAGGGCATCGACTT	ChIP/Copy number
GFP	R	GATGCCGTTCTTCTGCTTGT	ChIP/Copy number
MMERGLN	F	CGTAAGGACCCTAGTGGCTG	ChIP/RT-qPCR
MMERGLN	R	GCACTCACTCTTCTTCACTCTG	ChIP/RT-qPCR
MLV	F	GTAAAAACTCCACACTCGGC	ChIP/RT-qPCR
MLV	R	ACGATTCGGATGCAAACAGC	ChIP/RT-qPCR
RLTR4	F	CATACTTCTGCCCCAGCTAA	ChIP/RT-qPCR
RLTR4	R	CAGTAATCGGTGGTGAGGTC	ChIP/RT-qPCR
VL30int	F	TCAACAGGCCAGATGTATTGC	ChIP/RT-qPCR
VL30int	R	ACAAACTGGGAGGGGGAAT	ChIP/RT-qPCR
VL30u3i	F	AGATGTATTGCCAAACACAGG	ChIP/RT-qPCR
VL30u3i	R	AGGGGGAATGGGGAGGGAA	ChIP/RT-qPCR
VL30u3ii	F	GAACTCTTCCTCACCCCAGA	ChIP/RT-qPCR
VL30u3ii	R	GAGGAGGAGTTCAGGAATGC	ChIP/RT-qPCR
VL30u3iii	F	CTTTTCACCCCAAAACTCCTC	ChIP/RT-qPCR
VL30u3iii	R	CATCACTAGGGAGTTCTGCCA	ChIP/RT-qPCR
VL30u3iv	F	CCTCAAAATGACATTGCCAAA	ChIP/RT-qPCR
VL30u3iv	R	TTTCACAGGCTTATATAGTAAAACTC	ChIP/RT-qPCR

Table S1: Primer list (continued):

Target	F/R	sequence	application
IAP 5UTR	F	CGGGTCGCGGTAATAAAGGT	ChIP/RT-qPCR
IAP 5UTR	R	ACTCTCGTTCCCCAGCTGAA	ChIP/RT-qPCR
IAP1	F	CGTGAGAACGCGTCGAATAA	ChIP/RT-qPCR
IAP1	R	TTCTGGTTCTGGAATGAGGG	ChIP/RT-qPCR
IAP1-Mm_I	F	CTACCTGTGGACGTTGCCTT	ChIP/RT-qPCR
IAP1-Mm_I	R	CATCCATCTGCCACACCTGT	ChIP/RT-qPCR
IAP5	F	GTGCGGTCAGTCCTACGTAA	ChIP/RT-qPCR
IAP5	R	CCCGGAGCAGAAGTGAAAGT	ChIP/RT-qPCR
IAPLTR1	F	CAGTGCGCAGACTCATTCAT	ChIP/RT-qPCR
IAPLTR1	R	CTCGGCTCCTTCAAAGACTG	ChIP/RT-qPCR
IAPLTR3	F	GGCAAACCTCAGAAGGACAG	ChIP/RT-qPCR
IAPLTR3	R	CACATCTCTGCCCCATAGGT	ChIP/RT-qPCR
IAPLTR4	F	CAGCTGAAAGGCACAGACAA	ChIP/RT-qPCR
IAPLTR4	R	AAATAGGATCCGGGCCATAC	ChIP/RT-qPCR
IAP-D	F	TGAGAGAGGAGCGATCCCAA	ChIP/RT-qPCR
IAP-D	R	GACTGTCGGCTATGCTCTCC	ChIP/RT-qPCR
IAPEYI	F	TTGGTGGGCCACTCACTAAT	ChIP/RT-qPCR
IAPEYI	R	GTTTGAGCTCAGCCATGTCA	ChIP/RT-qPCR
IAPEy3	F	TGGTGTGGAAGATGCTGACC	ChIP/RT-qPCR
IAPEy3	R	CCCATCTCTGTTCGCCCATT	ChIP/RT-qPCR
IAPEz	F	GCTCCTGAAGATGTAAGCAATAAAG	ChIP/RT-qPCR
IAPEz	R	CTTCCTTGCGCCAGTCCCGAG	ChIP/RT-qPCR
MMERVK10C	F	CAAATAGCCCTACCATATGTCAG	ChIP/RT-qPCR
MMERVK10C	R	GTATACTTTCTTCTTCAGGTCCAC	ChIP/RT-qPCR
ETn-MusD	F	GTGCTAACCCAACGCTGGTTC	ChIP/RT-qPCR
ETn-MusD	R	CTCTGGCCTGAAACAACTCCTG	ChIP/RT-qPCR
RLTR45	F	TGCTTTTCCGACATGGTAAT	ChIP/RT-qPCR
RLTR45	R	AGTAACCCTGACCTGCTCCT	ChIP/RT-qPCR
MERVL	F	ATCTCCTGGCACCTGGTATG	ChIP/RT-qPCR
MERVL	R	AGAAGAAGGCATTTGCCAGA	ChIP/RT-qPCR
MuLV	F	GGCAGCCATACATACAGACC	ChIP/RT-qPCR
MuLV	R	TGGTCTGCATAGAAACAGCA	ChIP/RT-qPCR
LINE1	F	GATTACCAGATGGCGAAAGG	ChIP/RT-qPCR
LINE1	R	AGTGCTGCGTTCTGATGATG	ChIP/RT-qPCR
bis40nt(88)	F	GTT ATT TTG TAA GGT ATG GAA AAA	Bisulfite sequencing
bis40nt(727)	R	ACA AAC ACA AAT AAA TTA CTA ACC A	Bisulfite sequencing
bis40nt(382)	F	GAA ATG ATT TTG TGT TTT ATT TGA A	Bisulfite sequencing
bis40nt(715)	R	AAA TTA CTA ACC AAC TTA CCT CCC	Bisulfite sequencing
